# Effectively utilizing publicly available databases for cancer target evaluation

**DOI:** 10.1093/narcan/zcad035

**Published:** 2023-07-14

**Authors:** Daniel Croft, Puja Lodhia, Sofia Lourenco, Craig MacKay

**Affiliations:** Cancer Research Horizons, The Cancer Research UK Beatson Institute, Glasgow, G61 1BD, UK; Cancer Research Horizons, The Francis Crick Institute, London, NW1 1AT, UK; Cancer Research Horizons, The Francis Crick Institute, London, NW1 1AT, UK; Cancer Research Horizons, The Cancer Research UK Beatson Institute, Glasgow, G61 1BD, UK

## Abstract

The majority of compounds designed against cancer drug targets do not progress to become approved drugs, mainly due to lack of efficacy and/or unmanageable toxicity. Robust target evaluation is therefore required before progressing through the drug discovery process to reduce the high attrition rate. There are a wealth of publicly available databases that can be mined to generate data as part of a target evaluation. It can, however, be challenging to learn what databases are available, how and when they should be used, and to understand the associated limitations. Here, we have compiled and present key, freely accessible and easy-to-use databases that house informative datasets from *in vitro*, *in vivo* and clinical studies. We also highlight comprehensive target review databases that aim to bring together information from multiple sources into one-stop portals. In the post-genomics era, a key objective is to exploit the extensive cell, animal and patient characterization datasets in order to deliver precision medicine on a patient-specific basis. Effective utilization of the highlighted databases will go some way towards supporting the cancer research community achieve these aims.

## Graphical Abstract

**Graphical Abstract:**
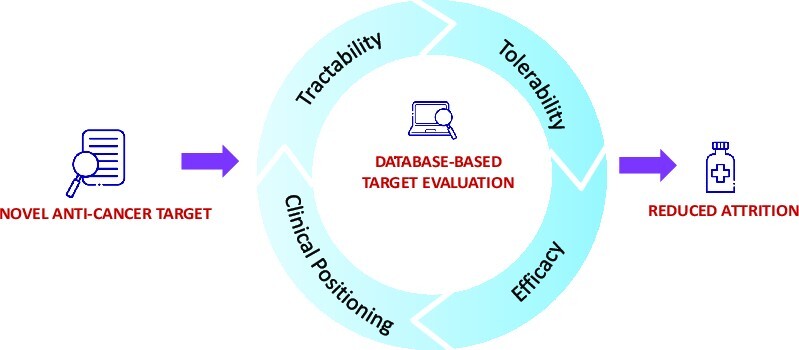

## INTRODUCTION

**Figure 1.**
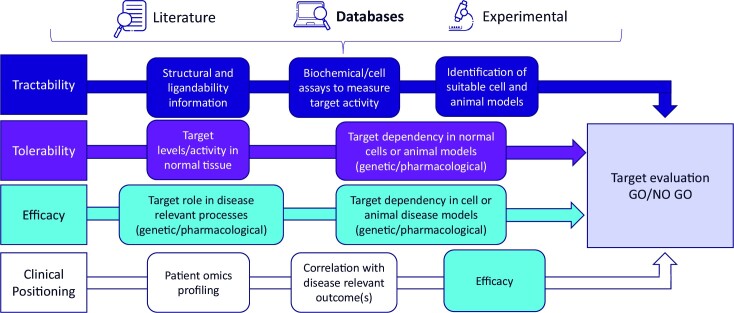
Target evaluation summary. Schematic diagram illustrating tractability, tolerability, efficacy and clinical positioning information that is required as part of a target evaluation assessment to enable decisions to be made regarding progression of a project into the full drug discovery process. Information can be obtained from a variety of sources, including online databases as highlighted.

Oncology drug discovery aims to develop therapeutic agents that modulate biological processes to inhibit progression of cancer in the clinical setting. Drug discovery is a resource-intensive process that can broadly be broken down into four steps: target identification and validation, hit identification and validation, lead identification and optimization, and clinical development (1). Each subsequent step is more resource intensive than the last, requiring significant financial investment. The average time for a new drug to go from the start of the process to approval is 10–15 years with costs exceeding $1 billion (2,3). Despite the significant resource involved, over 90% of new oncology agents do not become approved drugs, mainly due to lack of efficacy and/or unmanageable toxicity (4). It is therefore essential that sufficient efforts are invested in the evaluation of a novel target before a project progresses into drug discovery. The main aims of target evaluation (Figure 1) are to establish whether available information suggests that an agent modulating target activity is likely to be efficacious and tolerable in the clinical setting, identify the patient population to target (clinical positioning) and determine whether the project is technically feasible (tractability) (5).

As efforts to identify new therapeutic interventions in oncology have moved from broad-spectrum cytotoxic agents to selective agents against specific targets dysregulated in disease or agents that modify the tumour immune response (6), our need to understand target biology in different settings has drastically increased. The complex nature of cancer progression and the interplay between tumour intrinsic effects and interactions with the tumour microenvironment make it extremely challenging to generate all the information required for robust evaluation of a target. For novel targets, it is often the case that there is not a sufficient depth of literature available. However, in the post-genomics era, the extensive availability of omics datasets from cell lines, animal models and patient samples means that detailed information is available to support comprehensive target evaluation. As detailed in Figure 1, there are several sources for information that can inform tractability, tolerability, efficacy and clinical positioning assessments. One valuable source that we believe is currently under-utilized is the wealth of publicly available databases that can be mined to generate these data. Database mining can be used to support the cancer research community achieve two key objectives: The first is to ensure that targets are robustly evaluated prior to entering the drug discovery process, with the aim of reducing the high attrition rate at later stages. The second is to support the progression of targets that offer the opportunity to develop precision medicines on a patient-specific basis.

The wealth of available databases can, however, present a significant challenge. How do we gain an awareness of the databases, learn what information they can provide and understand their limitations? To help address these key questions, we highlight several key freely available databases and discuss how they can be utilized as part of a target evaluation. To demonstrate the utility of the selected databases, we present selected key examples of data outputs that can be obtained. We also discuss the limitations of the available databases and suggest additional information that would be of use to the cancer research and drug discovery communities. Finally, we demonstrate how the database outputs can be combined to evaluate a novel target and demonstrate how outputs can be used to predict challenges associated with clinical development.

## DATABASES

### Cancer cell line

The primary mechanism of action of the majority of approved anti-cancer drugs is to directly inhibit proliferation or to induce tumour cell death (7). Demonstrating cancer cell line target dependency for proliferation and/or viability therefore provides key validation for targets whose function in disease is primarily mediated through cancer cell intrinsic effects. In addition to predicting efficacy of target inhibition, output from cancer cell line profiling databases can be used to guide clinical positioning and support the identification of suitable clinically relevant cellular models in which to test compound efficacy.

The Cancer Dependency Map (DepMap) portal (https://depmap.org/portal/) is an initiative from the Broad and Sanger Institutes that houses large-scale RNAi and CRISPR screening from several sources with the aim of identifying intrinsic vulnerabilities of cancer cell lines. In addition, the DepMap portal also houses small molecule inhibitor screening data to identify pharmacological sensitivities across large cancer cell line panels, which we cover in the ‘Identification and profiling of tool compounds’ section.

Within DepMap, data from large-scale CRISPR (8,9) or RNAi (10–12) screens in cancer cell lines have been combined. The Chronos (13) or DEMTER2 (14) algorithms are used to normalize datasets to produce a single integrated output. Output is presented in the form of a ‘gene effect’ score where anything below 0 represents a loss of viability, with −1 being the median score for common essential genes. Data integration allows over 1000 cancer cell lines for CRISPR and over 700 cell lines for RNAi to be compared. Combining screening data from different primary screens that have been performed using distinct methodologies could result in variability across datasets, diminishing the statistical power to identify cell line-specific vulnerabilities. However, a comparative analysis between two of the largest contributors to the CRISPR screening dataset demonstrated that despite the differences in experimental set-up, there was a high degree of concordance between the studies in terms of both cell line-specific dependencies and predictive biomarkers identified (15), which increases confidence in the use of DepMap CRISPR screening datasets to support cancer target evaluation.

**Figure 2.**
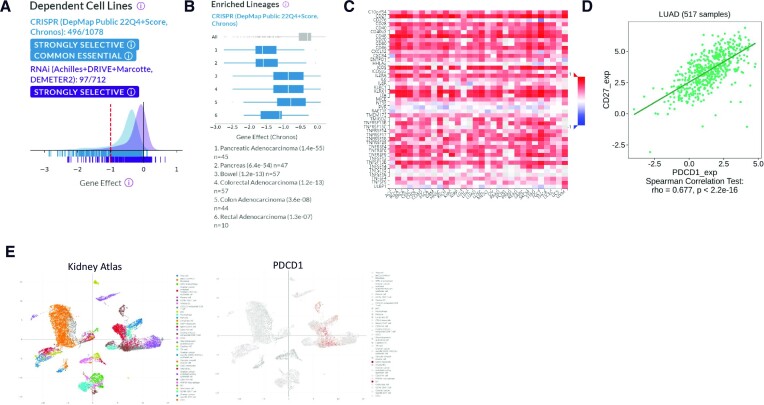
Cancer and immune cell database outputs. (**A**) Cell line dependency information for KRAS obtained from the DepMap portal. The gene effect score for CRISPR (light blue) and RNAi (dark blue) screening for ∼1000 cell lines each is plotted and a text summary is provided above the graph. Cell lines are classed as dependent on the target if the gene effect score is <−0.5 and the median gene effect score for a common essential gene is −1, as indicated by the dashed line. ‘Strongly selective’ dependency on KRAS indicates that there is a subset of the cell lines that are highly dependent on KRAS, but this is not common across the majority of cell lines tested. (**B**) Gene effect scores for CRISPR are plotted for all cell lines and then compared to the spread of gene effect scores for individual lineages. *P*-values are used to indicate statistically significant enrichment of target dependency observed within cell lines of pancreatic and colorectal origin relative to the pooled cell line panel. (**C**) The Tumor–Immune System Interaction Database (TISIDB) was used to calculate the Spearman correlation coefficient between expression of PDCD1 and gene expression of known immunostimulators across human cancers based on The Cancer Genome Atlas (TCGA) datasets. Data are presented as a heatmap with strong positive correlations indicated in red and negative correlations indicated in blue. (**D**) Individual boxes within the heatmap can be analysed in more detail as shown for the correlation plot of PDCD1 and CD27 expression in lung adenocarcinoma patients. (**E**) Deeply Integrated human Single-Cell Omics data (DISCO) scRNA-seq uniform manifold approximation and projection (UMAP) is shown for PDCD1 expression in the indicated immune cell subtypes distributed within the kidney atlas.

In addition to target dependency information, DepMap also houses genomic and metabolomic characterization from the Cancer Cell Line Encyclopedia (https://sites.broadinstitute.org/ccle/) (16–19). This includes mRNA expression, copy number and mutation status data, with recent efforts being made to expand this characterization to include mass spectrometry-based quantification of the proteome (20). Integration of this comprehensive omics dataset with extensive genetic and pharmacological screening gives the statistical power to make predictions to inform clinical positioning. This is exemplified by the DepMap output of dependent cell lines for the well-validated anti-cancer drug target KRAS (Figure 2A). It is well established that gain-of-function mutations in KRAS are prevalent in pancreatic, colorectal and non-small cell lung cancer (21) and act as a driver of tumour progression. The DepMap output for KRAS demonstrates that dependency on KRAS is strongly selective, with a clear ‘tail’ of strongly dependent cell lines (gene effect score ≤−1) observed with both RNAi and CRISPR screening (Figure 2A). Analysis of this dataset demonstrates that there is a statistical enrichment of KRAS dependency in cell lines of a pancreatic and colorectal origin identified from the CRISPR target dependency screens (Figure 2B), consistent with the high prevalence of gain-of-function mutations in these indications. Further evidence of the power of DepMap to identify selective dependencies on a target based on genomic features of cell lines comes from the observation that the WRN DNA helicase was identified as a selective vulnerability for microsatellite instable (MSI) cancer cell lines using CRISPR and RNAi screening data, now housed within DepMap (22). As a result, there is now significant commercial interest in developing selective WRN small molecule inhibitors for use in MSI tumours.

Cell Model Passports (https://cellmodelpassports.sanger.ac.uk/) is an integrated component of the DepMap that offers its own user-friendly portal with genomic and clinical characterization of >2000 cancer cell line models (23). The portal allows the user to search by cell line to identify all available information, including presence of driver mutations, mRNA and protein expression, copy number alterations and sensitivity to drugs tested by the Genomics of Drug Sensitivity in Cancer initiative (https://www.cancerrxgene.org/) (24). Integration within DepMap allows correlations between cell features and target dependency to be identified.

Synthetic lethality, where co-deletion/inhibition of two targets results in a loss of viability that is not observed upon perturbation of one target alone, is a strategy that offers the opportunity to achieve a therapeutic window due to limited effects on cells not bearing the cancer-specific alteration. The clinical relevance of this concept has been illustrated by the success of PARP inhibitors in BRCA1/2 mutant settings (25). There have been recent efforts to generate databases that can predict synthetic lethal interactions, such as SynLethDB (26), based on compiling synthetic lethal CRISPR screening data from literature. The power of these databases to identify novel targets is dependent on the quality and quantity of the compiled data and is currently limited by the scale of available synthetic lethality screening studies. In the coming years, we expect that these databases will have the potential to further inform clinical positioning strategies and identify novel targets that may only be revealed in a synthetic lethal setting.

Despite the power of cancer cell line databases, there remain several key limitations that should be considered when interpreting outputs. Depletion or deletion of a target is not necessarily recapitulated in full by inhibition of target activity, which is currently the most common modality of novel anti-cancer agents. Therefore, deletion/depletion data should be interpreted with caution, especially for targets that have multiple activities, as exemplified by the several known kinase-independent functions of protein and lipid kinases (27). Another key limitation is that cell screening data housed in DepMap have been generated under standard cell culture media conditions. It has become apparent that the composition of standard cell culture media conditions does not accurately recapitulate the *in vivo* environment and that utilizing physiologically relevant cell culture media can alter the metabolic profile of cancer cell lines in 2D and 3D growth conditions (28,29). Indeed, CRISPR screens performed in haemopoietic cell lines under physiological culture conditions result in the identification of specific gene dependencies not revealed in conventional culture conditions (30). This demonstrates that DepMap outputs will not always accurately reflect dependencies of cancer cell lines for certain targets, particularly those that play a role in metabolic processes, where dependency may be influenced by gene–nutrient interaction.

Cancer cell line growth in 3D conditions is believed to more accurately recapitulate the *in vivo* environment (31,32) and CRISPR screens comparing gene dependencies in 2D versus 3D conditions have identified 3D culture-specific vulnerabilities (33). Known cancer drivers and genes found to be heavily mutated in cancer were more likely to be identified by screening under 3D conditions, suggesting that there may be reduced target attrition at later stages of the drug discovery process, if 3D culture was used for novel target identification screens. A clear gap therefore exists for a database that houses large-scale CRISPR screening performed using 3D growth and physiologically relevant culture conditions.

Another key limitation of screening data from cancer cell lines is that using cancer cell lines alone is not an accurate reflection of the complex situation *in vivo*, where the tumour microenvironment, consisting of a range of cell types including stromal and immune cells, can play a key role in outcomes of therapeutic interventions. Lack of cancer cell intrinsic dependency on a target in this class would not therefore be indicative of response to perturbation *in vivo*. This is exemplified by zero cell lines being identified in DepMap CRISPR or RNAi datasets as being dependent on PD-L1 (https://depmap.org/portal/gene/CD274). Anti-PD-L1 antibodies have demonstrated efficacy in the clinical setting, where they act by enhancing the T-cell-mediated anti-tumour immune response (34).

A growing body of research highlights the role of the immune system in cancer initiation, progression, treatment and resistance to therapy (35). The interaction between malignant and immune cells is therefore an important consideration and several anti-cancer agents act by directly regulating the tumour immune response. Genome-wide CRISPR screens have been performed in immune cells such as macrophages and T cells (34–36) to identify factors required for cancer-relevant phenotypes, such as proliferation, viability and exhaustion. A database that compiled this information to cross-reference with cancer cell intrinsic dependency databases would be a step towards greater understanding of the impact of target inhibition in a more complex setting.

Once a target has been identified, database mining can also be used to identify interactors of the target of interest. Identifying known interactors can provide further mechanistic insight and lead to additional therapeutic opportunities, such as small molecule-mediated disruption of disease-relevant protein–protein interactions (PPIs). It is also possible that more tractable targets that play a similar role in disease progression can be identified through such efforts. The STRING database (https://string-db.org/) collates PPIs from several sources, including literature mining, computational prediction and databases of interactions identified experimentally (36). Several other PPI databases exist, and a thorough comparison of coverage has been reported (37), demonstrating that STRING has the highest coverage of experimentally verified PPIs and is updated frequently.

### Immune cells

The immune oncology field has been driven forward in recent years by the approval of therapeutic antibodies targeting CTLA4, PD-1 and PD-L1 (38). The success of such immune checkpoint inhibitors encouraged development of therapeutic agents against additional targets. However, the outcomes of clinical trials for novel immunotherapies have been underwhelming (39), highlighting the complexity of the tumour microenvironment and the need to better understand the cross-talk between tumour cells and infiltrating components of the immune system.

Understanding the role a target plays in regulating the tumour immune response is key for all aspects of target evaluation. A particular immune cell may be the most appropriate model in which to measure target activity and dependency. Clinical positioning may be more accurately guided by tumour extrinsic effects and a role in normal immune functions will inform tolerability assessments. It is therefore recommended that the databases highlighted in this section are utilized as part of a comprehensive target evaluation.

Gene expression data are the most abundant omics data available for both patient-derived samples and cancer cell lines and are a commonly utilized resource for researchers looking to understand genotype–phenotype relationships. TCGA, for example, has gene expression data from >10 000 patient tumour samples (see the ‘Clinical’ section). However, these data are derived from bulk RNA sequencing (RNA-seq) that does not differentiate between the various cell types, such as immune and tumour cells, present in a sample. To address this issue, various groups have derived deconvolution methods to give estimates of immune infiltration from bulk RNA-seq data, including Cibersort (40), Estimate (41), xCell (41), EPIC (42), quanTIseq (43), mMCPcounter (44) and TIMER (45).

The Tumor IMmune Estimation Resource (TIMER) (http://timer.cistrome.org/), developed by the Liu lab at the Dana Faber Institute, provides a portal that allows researchers to explore the infiltration of immune cells in TCGA tumour samples and correlate this with gene expression, mutation and copy number alterations. Infiltration scores calculated with the aforementioned deconvolution algorithms are available. However, cross-comparison of output from the different deconvolution methods is important as no single method is completely accurate and in some cases the infiltration scores can even be conflicting. Such data should therefore be used primarily to guide further experimental validation. TIMER also provides the functionality to determine the association between gene expression, immune infiltration and clinical outcome. While the data for TCGA samples are directly available via the TIMER portal, there is also the option for researchers to upload custom bulk RNA-seq data to analyse data generated elsewhere.

TISIDB (http://cis.hku.hk/TISIDB/) predicts response to immunotherapy by integrating datasets for a given target from multiple sources, including gene expression data, high-throughput CRISPR/shRNA screening to determine sensitivity to T-cell-mediated killing and literature mining of several thousand publications (46). When querying a target of interest, TISIDB presents associated information in a multi-tab format, which includes text mining results for evidence in the literature that links the target to immunotherapy response. The output also comprises omics data from clinical samples to identify correlations between pre-immunotherapy treatment target expression and immune-relevant cancer subtype or between target expression, mutation, copy number or methylation status with response to immune checkpoint inhibitors, abundance of tumour infiltrating lymphocyte subtypes, expression of known immunomodulators or chemokine expression. Like TIMER, TISIDB provides correlation data as an easy to visualize and interpret heatmap. For example, a heatmap is used to visualize the correlation of PDCD1 (encodes PD-1 protein) expression and known immunomodulators across a range of human cancers (Figure 2C). Red squares indicate positive correlation between PDCD1 expression and expression of named immunomodulators, which for a novel target could provide strong evidence of a potential link to the tumour immune response, to be followed up experimentally. This dataset can be further probed by visualization of correlations with specific immunomodulators within a cancer indication of interest, as illustrated by the correlation of PDCD1 and CD27 expression in lung adenocarcinoma (Figure 2D), as has been previously shown for breast cancer (47). TISIDB also provides the ability, within the ‘Immunotherapy’ tab, to identify target gene expression differences between responders and non-responders to immune checkpoint inhibition from patient samples. However, low patient numbers are a general feature of studies housed within TISIDB, which limits the ability to identify statistically significant differences. Overall, TISIDB provides a comprehensive overview that supports detailed evaluation of the potential role of a novel target in the cancer immune response.

TIMER and TISIDB have been utilized effectively as tools to identify correlations between target expression (48,49) or DNA methylation status (50) and tumour immune cell infiltration and prognosis. This demonstrates the power of such correlative analysis to identify biomarkers or novel targets that regulate the tumour immune response.

The gene expression datasets used by TIMER and TISIDB are primarily from bulk RNA-seq experiments, and the deconvolution methods employed only estimate the heterogeneity of the cell types present within the sample. This has led to a shift towards single-cell RNA-seq (scRNA-seq) to give accurate expression profiles of individual cells. Until recently, scRNA-seq datasets have been notoriously difficult to access and analyse without considerable programming knowledge. Although publications require data to be deposited in repositories such as Gene Expression Omnibus (51) and the Database of Genotypes and Phenotypes (52), there is little consistency in formatting or even at what stage of processing the data are deposited, which complicates data extraction and analysis. Several groups have produced online portals to facilitate easier access to scRNA-seq datasets including the Single Cell Expression Atlas (https://www.ebi.ac.uk/gxa/sc/home) by EMBL-EBI (53) and CellxGene (https://cellxgene.cziscience.com/) from the Chan Zuckerberg Biohub project (54). The datasets provided by Single Cell Expression Atlas are limited, with only 131 human and 111 mouse experiments available at the time of writing. The tool allows expression of a gene of interest to be projected on to the UMAP (55) or t-stochastic neighbourhood embedding (56) plots (the two main graphical methods for scRNA-seq data visualization) of a chosen dataset. However, metadata annotations can be variable, with some datasets critically missing cell type assignments. CellxGene houses a much larger collection of datasets with >600 human studies currently available. The explore function provides extensive options for annotating the UMAP of each dataset with study parameters and author cell assignments but also importantly includes quality control metrics such as mitochondrial fraction and unique molecular identifier counts for users to confirm data quality. As with other tools, specific genes can be plotted on the UMAP, but CellxGene also offers a gene set function that allows users to plot multiple genes together, which is useful for exploring gene signatures.

An alternative portal for exploration of scRNA-seq data is DISCO (https://www.immunesinglecell.org/) (57). DISCO contains a large collection of human scRNA-seq datasets integrated into tissue-specific ‘atlases’. Each atlas can be queried by gene ID with outputs given as UMAPs or violin plots by cell type. Datasets can also be queried in a pan-tissue manner, which can be informative when evaluating a target for which the appropriate tissue context may not yet be known. DISCO allows visualization of target expression in immune cell types across all atlases as a violin plot to give an overview of distribution of target expression. Exploring the different atlases leads to the interactive UMAP tool that can be used to view the expression of a target gene at a cellular level, as shown for PDCD1 in a kidney atlas (Figure 2E) (58). These data can inform the selection of appropriate immune cell types in which to study target biology and direct clinical positioning efforts. There are several disease-specific atlases available, including for pancreatic ductal adenocarcinoma, ovarian cancer and triple negative breast cancer, which have the added functionality of being able to perform disease versus normal comparison for a gene of interest. Expansion of cancer-specific atlases in the coming years will further enhance the utility of DISCO for cancer target evaluation. DISCO also offers a ‘FastIntegration’ tool that allows specific datasets of interest to be integrated and analysed together. Such capabilities have until now not been possible without programming knowledge and use of tools such as the R package Seurat. Data from DISCO can also be downloaded as a Seurat object to facilitate more complex analysis if required.

### Mouse models

An integral component of a target evaluation is to determine whether there is sufficient validation of target dependency via genetic or pharmacological methods in pre-clinical models that accurately model the complex nature of cancer in the physiological setting. This complex physiology includes understanding the impact of target modulation in a model that includes a proliferating tumour and components of the tumour microenvironment and that represents the inherent heterogeneity of human disease. Demonstration of efficacy in clinically relevant models is also used to guide clinical positioning strategies. Another key aim of target evaluation is to predict toxicity associated with target inhibition, using genetic or pharmacological methods. Mouse models have been used extensively for each of these aims and are routinely used to predict compound efficacy and toxicity in a clinical setting. Several databases compiling mouse datasets across multiple models provide different platforms that can be mined to both obtain available information and identify suitable models that can be used experimentally.

The International Mouse Phenotyping Consortium (IMPC) (https://www.mousephenotype.org) has been established between 21 research institutes with the aim of creating murine knockouts for every protein-coding gene within the mouse genome (59,60). Knockout generation and characterization are all performed within the consortium. Standard pipelines for phenotypic characterization are applied, enabling valid comparison between all knockout models. If homozygous knockout mice are viable, then extensive phenotyping of the early adult will take place between 9 and 15 weeks, or if not viable, then heterozygotes will be characterized, and the stage of embryonic lethality of the homozygotes will be determined. Querying IMPC by gene name will bring up a link, if available, to a graphical representation of the overall phenotypic characterization where 20 different phenotypic outputs are coloured to represent significant differences to wild type, no significant difference or not tested. The full details of the phenotypic characterization and an analysis of body weight can also be found within this page. Such phenotypic characterization data can be used to flag potential tolerability concerns associated with loss of a target protein that may be previously unknown. Since unmanageable toxicity is a key reason for attrition of targets during the drug discovery and clinical development process, it is essential that potential liabilities are flagged as part of a target evaluation. Knockout mice, embryonic stem cells or targeting vectors can also be purchased directly through this portal.

It is, however, important to note that knockout mouse data are a rather crude way to evaluate potential toxicity liabilities of a therapeutic, such as a small molecule inhibitor. Many proteins have multiple functions and target knockout will ablate all functions, resulting in a phenotype that may not be representative of therapeutic intervention. Key parameters for toxicity are the pharmacokinetic (PK) and pharmacodynamic (PD) properties of a therapeutic agent that are also not reflected by whole body or tissue-specific target knockout. Toxicity may also be driven by off-target effects of a therapeutic agent, which will not be predicted by target-directed evaluation.

The Mouse Models of Human Cancer Database (MMHCdb) (http://tumor.informatics.jax.org/) is a manually curated resource of several types of murine cancer models hosted by The Jackson Laboratory with funding from the National Cancer Institute (NCI) (61). MMHCdb is part of the Mouse Genome Informatics consortium, first released in 1998 as the Mouse Tumor Biology Database (62). The database houses data from over 46 000 models from nearly 7000 different cohorts of mice. Extensive efforts have been made to provide curated, consistent data to inform selection of clinically relevant models. Data are extracted from literature or submitted directly by individuals or large-scale research initiatives. The database bridges the historical gap around gene and strain nomenclature standards from diverse sources.

The three types of mouse models with available information within MMHCdb are inbred mouse models, genetically engineered mouse models (GEMMs) and patient-derived xenografts (PDXs). Information available for inbred mouse models includes an interactive graphical summary of the characteristic cancers observed in over 700 different inbred mouse strains. The Tumor Frequency Grid tool displays the frequency of spontaneous tumours across the different inbred strains. GEMMs are generated by introduction of murine equivalents of human cancer-associated mutations and can be used to study tumour initiation, progression and response to therapy (63). However, as illustrated by strain comparison using the Tumor Frequency Grid, the genetic background in which GEMMs are developed can have an impact on the observed phenotype. It is therefore essential that the influence of genetic backgrounds is taken into consideration when selecting appropriate models for transplantation or GEMM generation or when interpreting study data. To support model selection, the MMHCdb search function allows queries by gene type, cancer type and mouse strain to identify all associated studies and provide further information on tumour onset, pathology and sites of metastasis.

PDX models are generated by implantation of human tumour tissue in an immunodeficient or humanized mouse. In collaboration with EMBL-EBI, MMHCdb co-developed the PDX Finder resource that serves as a global catalogue of PDX models (64). The PDX Finder tool has since grown to include cancer cell line and organoid models and is now available as a stand-alone database, Patient Derived Cancer Models Finder (https://www.cancermodels.org), that can be used to identify clinically relevant patient-derived model systems for a given disease area and explore associated characterization. However, the majority of PDX models available within the MMHCdb are from the immunodeficient NSG host strain and therefore will not provide insight into potential interactions with the tumour immune response.

Syngeneic mouse models, where murine tissue or cell lines are transplanted into immune competent mouse models, allow the study of the tumour immune response in a complex setting. The Tumor Immune Syngeneic MOuse (TISMO) (http://tismo.cistrome.org/) database hosts datasets from 137 public syngeneic mouse model studies, comprising over 1500 samples from 68 different models (65). These models, however, do not cover all cancer indications, with brain cancers, for example, having no representative models within TISMO. In addition to manually curated model characterization, including details of cell line genotype and cancer type, mouse genetic background and implantation site, TISMO provides interactive visual interfaces to explore datasets for gene expression, immune cell infiltrate and response to therapy, in both treatment naïve and immune checkpoint blockade treated models.

There are several specific features of TISMO worth highlighting. The first is the ‘Pathway’ tab that allows comparison between different biological pathways, from KEGG, GO cellular compartment, WikiPathways, GO molecular function, GO biological process, Reactome and MSigDB C7 immunologic signature, across different tumour models and between pre- and post-treatment with immune checkpoint inhibitor treatment. These data can provide evidence that a target of interest plays a role in the response to specific therapies in mouse tumour models with specific genetic backgrounds. TISMO also allows upload of user gene sets for custom analysis, which is a powerful feature when evaluating the role of a novel target in the tumour immune response. Within the ‘Infiltrate’ tab, users can compare immune cell infiltration levels across different tumour models, between pre- and post-treatment, and between immune checkpoint inhibitor responders and non-responders. As discussed in the immune cell profiling section, immune cell infiltrates are not measured directly but rather estimated using deconvolution algorithms and therefore the data output should be used to guide further experimental validation. The main drawback compared to human databases is the limited number of immune cell types and signatures available to be assessed. TISMO currently only allows analysis of CD8^+^ T-cell, CD4^+^ T-cell, macrophage, dendritic cell, B-cell and neutrophil infiltrates.

Syngeneic mouse models are extensively used in immune oncology studies, generating an ever-expanding volume of gene expression, immune infiltration and treatment response data. The field has suffered from a lack of systematic collection and variation between analysis methods, which is being addressed by databases such as TISMO. TISMO is currently the only database with a comprehensive collection of datasets from syngeneic mouse tumour models. This database has also recently been used to support machine learning on syngeneic mouse tumour profiles to model clinical immunotherapy responses (66). Additional features that would enhance the utility of TISMO would be to allow the upload of propriety databases for analysis, enable correlation assessments between gene expression profiles and immune infiltration levels, and include available scRNA-seq datasets.

### Identification and profiling of tool compounds

Small molecule chemical probes are used in drug discovery as an orthogonal approach to genetic techniques in cellular and animal models in order to predict efficacy, assess target-related toxicity and explore target biology (Figure 1) (67). These tool compounds are usually small molecule inhibitors, but can be receptor antagonists, receptor agonists or other modulators, such as proteolysis-targeting chimeras (PROTACs; see below). Biological agents such as therapeutic antibodies can also be used for similar aims, but for the purposes of this review we focus on pharmacological agents.

The use of non-selective tool compounds that are unsuitable for biological studies is common and resulting data can misinform target evaluation. An example of a non-selective compound that is still widely used is the non-selective PI3 kinase inhibitor LY294002 (68). Online resources have therefore been developed to help researchers select and use the best tool compounds for their studies (69). Such information can also be used to evaluate how informative existing literature or screening data utilizing compounds may be.

Probe Miner (https://probeminer.icr.ac.uk) was developed by the Institute of Cancer Research to objectively identify the most suitable tool compounds (70). It uses chemical and bioactivity data from large-scale public databases such as BindingDB (https://www.bindingdb.org/) and ChEMBL (https://www.ebi.ac.uk/chembl/) to assess over 1.8 million compounds (70–73). Probe Miner integrates fitness scores for cellular potency, target selectivity, permeability, structure–activity relationships, inactive analogues and pan-assay interference to automatically rank compounds for a particular protein target (70). Probe Miner displays a distribution of the top 20 rated chemical probes together with a compound viewer containing the chemical structure and a radar plot highlighting the strengths and limitations of each chemical tool. A direct link is also provided to common probes recommended by the Chemical Probes Portal so that the user can access guidance for best use of these reviewed compounds. Moreover, Probe Miner has identified high-quality compounds that have been prioritized for future appraisal by the Chemical Probes Portal (see below). Compounds that do not meet the minimum requirements (potency <100 nM; selectivity >10-fold against any other protein; and permeability, effects in cells at <10 μM) are flagged with a recommendation to use with caution or avoid when better compounds are available.

The Chemical Probes Portal (https://www.chemicalprobes.org) is a manually curated online resource for selecting tool compounds (67,69). It currently contains over 500 compounds that encompass >400 protein targets from 100 protein families. These compounds have been evaluated by chemical probe experts, who provide recommendations on the best available compounds, together with guidance on concentrations and conditions for use in cellular assays and *in vivo* models. Where available, the portal will highlight any inactive compound analogues and orthogonal compounds that can be used to confirm that observed phenotypes are target engagement dependent. The portal contains links to primary literature references, vendor websites and gene databases, and highlights flawed or outdated ‘historical compounds’ that researchers should avoid. For example, LY294002 is described in the Chemical Probes Portal as a ‘historical compound, not to be used as a selective chemical probe for a specific target’.

Additional databases that can be used to access molecular information for approved drugs and investigational compounds are DrugBank (https://go.drugbank.com/) (74) and the Structural Genomics Consortium (SGC) (https://www.thesgc.org/chemical-probes) (75). Using DrugBank to search by target links to known agents with activity towards that target, while searching by drug links to a wealth of information for that specific agent. This includes chemical structure, pharmacology, known drug–drug interactions, chemical properties and links to references for further information. DrugBank is freely available to use for non-commercial applications, but commercial use requires a licence. The SGC is a global partnership between academia, industry and funding agencies with one of the key aims being to create and characterize chemical probes that are made freely available with no restrictions on use. The SGC portal lists available chemical probes, sorted by target protein class. Clicking on the probe of interest links to all available information on probe properties, recommends a chemically similar negative control probe and provides a link to request the probe(s) of interest. Increased awareness and use of databases such as Probe Miner, the Chemical Probes Portal, DrugBank and the SGC will reduce the use of unsuitable tool compounds that can misinform target evaluation and promote the best practice of utilizing chemically similar negative control compounds to confirm on-target phenotypes.

PROTACs have become an increasingly attractive strategy for targeting ‘undruggable’ proteins (76,77). PROTAC molecules can exploit all surface binding sites and are not reliant on binding in a deep hydrophobic pocket or active sites to modulate target protein activity (78). PROTACs are heterobifunctional molecules that contain a warhead small molecule that binds the protein of interest, connected via a linker molecule to a small molecule E3 ligand that recruits an E3 ubiquitin ligase to degrade the bound target protein. PROTAC-DB (http://cadd.zju.edu.cn/protacdb/) is an online public database that collates currently described PROTAC molecules (79) and can be queried by target, compound name/ID or chemical structure. Output is presented as a datasheet showing 2D compound structures (divided into warhead, linker and E3 ligand), biological activities (degradation capacity, binding affinities and cellular activities) and calculated physicochemical properties. The database also utilizes a computational method (PROTAC-Model) to generate predicted ternary complex models for PROTACs that exhibit good degradation capacity (80). PROTACs can be a useful research tool to provide efficacy, tolerability or clinical positioning information. New modalities such as degrader approaches can also influence tractability assessments and allow targets previously deemed to have poor tractability to be revisited.

Targeted cancer therapies act by perturbing specific molecular pathways in tumours. However, analysis of the genomes from specific tumour types has shown that tumours are highly heterogeneous and that this heterogeneity can often explain varied patient responses to targeted therapies. This broad genetic heterogeneity is also observed across cancer cell lines (16). The Broad Institute and Harvard University have developed a novel screening technology called Profiling Relative Inhibition Simultaneously in Mixtures (PRISM) that enables simultaneous high-throughput drug screening in large panels of genetically characterized cell lines (81). This method allows pooled screening of cell lines labelled with unique DNA barcode sequences. Barcode abundance is used to generate cell line sensitivity signatures by comparing treatment to control conditions. Predictive models can identify molecular features that correlate with PRISM sensitivity profiles. The PRISM repurposing dataset (https://depmap.org/repurposing) is available on the DepMap portal and contains viability data generated using the PRISM multiplexed cell line assay to screen 578 cell lines with the Broad Repurposing Library (4518 compounds) (82). Approximately three quarters of the library compounds are approved clinical compounds or in clinical development, with the remainder consisting of tool compounds. This repurposing screen identified tepoxalin, a dual cyclooxygenase and lipoxygenase inhibitor, that selectively killed cell lines with elevated expression of the multidrug resistance protein, MDR1 (82). Expanding the PRISM drug repurposing resource to cover more compounds and cellular models would support repurposing of existing drugs into future cancer therapies. The output can also inform clinical positioning strategies for novel targets by identifying cell line features associated with sensitivity to tool compounds.

### Clinical

A key aim of target evaluation is to identify a clinical positioning strategy for a compound developed against a specific target. Patient omics profiling information, including gene mutation or target expression profiles, can be correlated with disease-relevant outcome(s) such as patient survival to refine this clinical positioning strategy, with the aim of delivering precision medicine. A clinical positioning strategy can also inform the selection of relevant model systems for efficacy predictions or compound testing. In this section, we describe key databases that house patient omics and survival data and discuss how these can be utilized.

The combination of cost-effective next-generation sequencing together with large-scale cancer genomic efforts, such as TCGA and the International Cancer Genome Consortium (ICGC), meant that online platforms were needed to integrate the ever-increasing datasets generated and make them readily accessible to the wider cancer research community. TCGA was initiated in 2006 as a joint effort between the NCI and the National Human Genome Research Institute to create a comprehensive ‘atlas’ of cancer genomic profiles by cataloguing cancer-causing genome alterations in the most prevalent human tumour types (83). During the subsequent 16 years, the initiative has generated multi-omics data, including gene expression, DNA mutation, copy number variant and DNA methylation, from over 20 000 primary cancer and matched normal samples across 33 cancer types (84). TCGA data can be accessed through the Genomic Data Commons data portal (https://portal.gdc.cancer.gov/). The portal provides different navigation options for browsing available datasets to view summaries of data for each project, explore data at the case, gene and mutation levels, or compare different cohorts or clinical variables of a specific cohort. One limitation for cancer versus normal comparisons from the TCGA is that the number of samples from adjacent normal tissue is often far lower than that for tumour samples, which reduces the statistical power of the analysis. An alternative non-tumour gene expression resource that can be used for comparison purposes is the Genotype-Tissue Expression project (https://gtexportal.org/home/) that has gene expression data for 54 normal tissue types from close to 1000 individuals.

**Figure 3.**
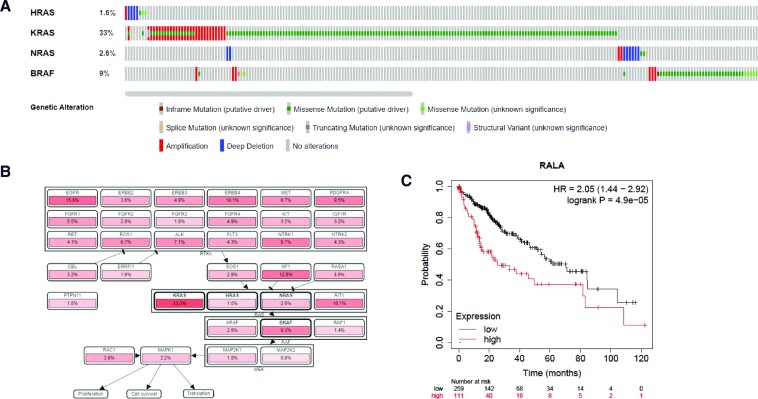
Clinical datasets. (**A**) The output from cBioPortal analysis of HRAS, KRAS, NRAS and BRAF genetic alterations found in a lung adenocarcinoma TCGA study. The overall prevalence of genetic alteration is indicated by a percentage for each gene and then further details of the type of alteration are represented by colour. Each vertical line of rectangles represents a single patient, so co-occurrence or mutual exclusivity of genetic alterations can be visualized, as indicated in this example by a general trend towards lack of co-occurrence of genetic alterations between the queried genes. (**B**) cBioPortal output using the same input query as panel (A), from the ‘Pathways’ tab. A schematic of the signalling pathway within lung adenocarcinoma is shown together with a genetic alteration frequency for each functionally linked gene. (**C**) A Kaplan–Meier plot from the Kaplan–Meier Plotter (KMplot) database demonstrating the correlation between high expression of RalA and reduced overall survival in hepatocellular carcinoma. Hazard ratios and logrank *P*-values are shown to demonstrate whether there is a statistically significant correlation observed.

The cBio Cancer Genomics Portal (https://www.cbioportal.org/) was developed at the Memorial Sloan-Kettering Cancer Center to enable the visualization and in-depth analysis of multi-omics patient data for various types of cancer (85,86). It houses data from the entire TCGA Pan-Cancer Atlas, comprising over 10 000 samples (87) as well as additional data from over 200 published studies with almost 70 000 patient samples that have been curated to ensure there is no redundancy between the studies. Users can query selected cancer studies to visualize the available omics data for single or multiple genes across patient samples. For example, querying the TCGA lung adenocarcinoma Pan-Cancer Atlas study for omics data pertaining to RAS pathway members generates a series of reports, including a summary of genomic alterations (Figure 3A). These data demonstrate that KRAS, HRAS, NRAS and BRAF gene alterations are mutually exclusive, pointing to the shared functional relationship between pathway members. This can then be explored further using the ‘Pathways’ tab that provides a schematic of signalling pathway(s) and functionally linked proteins to the user’s target query, together with details of alteration frequency for all related targets (Figure 3B). Mutual exclusivity of mutations in cancer can be used to identify vulnerabilities that can be exploited therapeutically, such as the observation that cyclin E1 amplification is mutually exclusive with BRCA1 mutation in high-grade serous ovarian cancers and that BRCA1 is selectively required for survival of cyclin E1 amplified cells (88). Such studies demonstrate the power of this analysis to identify clinical positioning opportunities.

Although the previously described databases focus on mRNA expression levels in cancer, the vast majority of anti-cancer drugs are designed against protein targets and changes in protein activity are the major driver of cancer progression. The NCI’s Clinical Proteomic Tumor Analysis Consortium (CPTAC) (https://proteomics.cancer.gov/programs/cptac) utilizes large-scale mass spectrometry-based methods to characterize the proteome of patient samples. This initiative was built on transcriptomic data from TCGA to characterize colorectal, breast and ovarian cancer samples (89–91). These initial studies demonstrated that mRNA levels do not correlate accurately with protein levels and that proteomic analysis could be used to further refine patient stratification and identify novel therapeutic targets. The CPTAC dataset has since expanded into several other indications and now comprises proteomic characterization of over 2000 patient samples (92–97). In addition to measuring protein levels, proteomic datasets also include information on post-translation modifications (PTMs). These data have been used to identify downstream targets of KRAS in pancreatic cancer (95) and to suggest that inhibition of Rb phosphorylation could be a viable therapeutic strategy in colorectal cancer (98). Recent re-analysis of CPTAC datasets using more powerful cloud computing methods has identified PTMs that were present at lower levels than could be previously identified and from a wider range than were previously considered (99), suggesting that the full potential of the CPTAC datasets is yet to be realized.

For the purposes of cancer target evaluation, the University of ALabama at Birmingham CANcer data analysis portal (UALCAN) (http://ualcan.path.uab.edu/analysis-prot.html) (100), a user-friendly web portal to analyse CPTAC datasets, houses proteomic data obtained from analysing 2002 patient samples from 17 separate studies (101). The portal allows determination of target protein levels and phosphorylation state across a range of cancer types in comparison to adjacent normal tissue. It can also be used as a user-friendly method of accessing TCGA gene expression and clinical survival data.

The major limitation of current proteomic databases for target evaluation is the limited sample size compared to that available for transcriptomics. Mining datasets comprising hundreds, or low thousands, of patient samples spread across several indications reduces the statistical power to detect significant alterations in protein level and/or PTM status within a single target indication.

An alternative initiative to map the abundance of human proteins across human tissues using antibody-based imaging and mass spectrometry approaches is the Human Protein Atlas (https://www.proteinatlas.org/). The Human Protein Atlas began by characterizing the expression and localization of 700 proteins across 48 normal human tissues and 20 cancer types using tissue microarrays (102) and has progressed to providing expression and localization data for over 16 000 proteins in all major tissues and organs (103). An extension of this initiative is the Human Pathology Atlas (104), which houses mRNA (from TCGA) and protein (from antibody-based imaging) levels for several cancer types. This dataset can be used as independent confirmation of data from CPTAC and knowledge of target protein levels in normal tissue may flag key tissue-specific or general housekeeping functions that could inform tolerability assessments. A future expansion of the Human Protein Atlas that would be of interest would be to build on the PTM analysis of CPTAC and use validated PTM-specific antibodies for cancer-associated proteins to identify and characterize PTM events that could be targeted in cancer.

cBioPortal, TCGA and the Human Protein Atlas datasets allow correlation of omics data with available patient data, providing evidence that target expression and/or mutation are associated with clinical outcome. A simple, user-friendly portal that houses manually curated datasets from the Gene Expression Omnibus (https://www.ncbi.nlm.nih.gov/geo/info/overview.html), the European Genome–Phenome Archive (https://ega-archive.org/) and TCGA is also available: The KMplot web tool (https://kmplot.com/analysis/) allows users to upload their own datasets or mine available miRNA and mRNA expression data and correlate with patient outcome (105). The mRNA expression data from RNA-seq analysis, for example, have data for >7000 patient samples across all indications correlated with overall survival. KMplot also allows the user to further restrict the analysis by cancer subtype, including sex, grade, mutation burden or enrichment of specific immune cell subtypes. The output is presented as a Kaplan–Meier survival plot for high/low expression of the target of interest together with a hazard ratio and logrank *P*-value to determine statistical significance. RalA is a GTPase that functions downstream of KRAS. KMplot has data from 259 patients classified as having low RALA expression and 111 with high RALA expression. Unrestricted analysis demonstrates that there is a statistically significant correlation between high RALA expression and reduced overall survival in hepatocellular carcinoma (Figure 3C), consistent with published data (106). Data demonstrating that high target expression correlates with poor outcome in a specific disease setting can inform clinical positioning assessments.

### Comprehensive target evaluation databases

There are now several open-source comprehensive databases that allow researchers to identify and prioritize potential drug targets for further study (107–109). Such databases aim to integrate information from several of the databases described above into a single easy-to-use platform.

The Open Targets Platform (https://platform.opentargets.org/) was developed as a public–private partnership between several European research institutes and pharmaceutical companies (107). It integrates data from 22 public sources and scores target–disease associations based on information regarding the target, disease, mutation, known drugs, animal models and scientific literature. The Open Targets Platform can be queried by target, drug, specific disease or phenotype (110,111). Searching by target generates a graphical visualization illustrating disease associations grouped by therapeutic area, together with a separate profile page of available information for the target. This profile includes details on known drugs (investigational or approved) for the target, reported safety effects, chemical probes, target expression (RNA and protein), molecular information and pathways, and phenotypes linked to mouse homologues. Also included in the profile page is a target tractability assessment for small molecule, antibody, PROTAC and other therapeutic modalities. Searching on a specific disease presents a list of targets associated with that disease that can be prioritized for further appraisal. Querying the platform for a specific drug shows its mechanism of action, investigational and approved indications, clinical trials, pharmacovigilance and all scientific literature associated with that drug.

TargetDB (https://github.com/sdecesco/targetDB) is a resource that allows users to rapidly query multiple public databases and generate an integrated summary of available information for a target (108). It is distributed as a Python package with SQLite database and can be downloaded by following the installation instructions on GitHub. TargetDB uses data obtained from public databases and sources such as ChEMBL, Open Targets, Protein Data Bank (PDB), PubMed, the Human Protein Atlas and UniProt (108). When queried for a single gene target, it generates an Excel file containing numerous worksheets with different target information: general information plus a spider plot summary of available data; PubMed search results listing the 500 most recent publications; disease information showing protein and GWAS associations; disease areas and association scores; protein expression; mouse genotypes and associated phenotypes; isoforms; observed variants and mutants; and a list of available crystal structures for the target. Also included is an analysis of potential small molecule binding pockets present in the target together with a ligandability score. Commercially available bioactive compounds are also listed with SMILES strings, target affinities and links to vendor websites. TargetDB allows queries for multiple targets using the list mode function to generate a report containing information for each target, or spider plot mode that outputs target information as a graphical representation.

The canSAR knowledgebase (https://cansar.ai/) is the largest publicly available online resource for translational cancer research and drug discovery (109,112). It was first released in 2011 (113) by the Institute of Cancer Research and has recently received major updates (109,112). This new edition integrates multi-omics patient and cell line data (ICGC and TCGA) with genetic mutation and dependency data (DepMap). The annotated human proteome (UniProt Swiss-Prot) together with data on protein 3D structures (PDB and AlphaFold), PPIs (IMEx Consortium and others), medicinal chemistry (ChEMBL and BindingDB) and clinical trials (ClinicalTrials.gov) is used by machine learning algorithms to assess ligandability of the target. Searching the canSAR knowledgebase by target takes the user to a dashboard that includes molecular synopsis pages for ligandability, signalling, disease types, experimental tools, features and chemical tools associated with the target. The ‘Disease types’ page shows a word cloud of cancer indications that are sized according to a cancer–target association score. There are clinical, mutation, copy number, expression and combined molecular scores available for each target with the score corresponding to how strong the link is between that particular target feature and the specified cancer target indication. This is a particularly powerful feature that allows previously unknown associations between the target and a disease setting to be identified. The ‘Ligandability’ tab provides a top line summary of areas of the protein associated with druggable cavities, a link to associated structural data and links to any approved or investigational drugs and chemical tools that can be used. canSAR labels associated chemical tools as ‘recommended’ or ‘acceptable’, depending on selectivity profiles, and provides direct links to the Chemical Probes Portal and Probe Miner for additional information. The ‘Experimental Tools’ synopsis page includes information on expression systems for expressing active target protein, known target engagement biomarkers and cell lines ranked on target expression, genetic dependencies and chemical dependencies identified with tool compounds known to act on the target. Additional features available for each target include a target gene family cladogram, target interactome and association of target mutation or expression with cancer indication-specific immune subtypes as defined by Thorsson *et al.* (114).

The comprehensive databases described above offer platforms that can quickly provide an overview of a wide range of available datasets for a given target in cancer and should be considered valuable tools for cancer target evaluation. However, no single platform currently covers all the datasets and tools highlighted in this review and therefore outputs from the various databases described should be considered together as part of a comprehensive target evaluation. The individual databases can also offer additional features or flexibility in data analysis that may provide further information.

### Artificial intelligence in drug discovery

Although not the focus of this review, is it impossible to ignore the growing interest in artificial intelligence (AI) and the potential utility across all stages of the drug discovery process. The area has been covered by several recent comprehensive reviews (115–117) that discuss the potential of AI to increase the efficiency of the drug discovery process in target identification, protein structure and druggable site predictions, *de novo* small molecule design and predictions of drug toxicity. We will therefore only briefly cover applications of AI that we see having a clear impact in the immediate future.

AI offers the opportunity to rapidly integrate the extensive datasets from multiple resources, such as those described in this review, to either identify novel targets or evaluate proposed targets in an unbiased manner (116). In the coming years, omics datasets are only to get larger and more complex. AI offers the potential to integrate and interpret complex outputs from multiple sources to produce unbiased outputs. Indeed, there are several drug discovery companies that currently utilize AI platforms for target identification that have assets in clinical development (118).

As described in previous sections, toxicity is a key reason for attrition of anti-cancer agents during the clinical development process. Current methods to predict toxicity, including evaluating mouse knockout phenotypes or comparing expression or target dependency in cancer versus cells of a non-malignant origin, have limited utility and make it challenging to effectively predict tolerability as part of a target evaluation. This is a key area of importance for the field as more accurate toxicity predictions may better predict the likelihood of clinical success. A potential application of AI will be to make more accurate predictions of toxicity based on predictions of compound PK/PD properties and off-target effects together with liabilities associated with specific chemical characteristics. A recent comprehensive review highlights the current status of toxicity prediction models and challenges associated with development in this area (119). A gold-standard accessible tool in this space will be of immense importance to support the design of compounds with minimal toxicity risks.

The final AI application we wish to highlight is in protein structure predictions. Structural information for a target can be used to identify small molecule binding pockets and to guide compound design in a rationale manner. Available structural information is therefore a key factor in tractability assessments. Currently, the Worldwide Protein Data Bank (http://www.wwpdb.org/) is the primary source for such information as the main repository for protein structural data (120). However, coverage of the human proteome is not complete and there may not be available information for a novel target. The AlphaFold Protein Structure Database (https://alphafold.ebi.ac.uk/), which has been developed by DeepMind from Google, uses AI to predict protein structural data from primary protein sequence, with almost complete coverage of the human proteome. AlphaFold predictions have been evaluated and found to correlate reliably with experimental data (121–125). Structural enablement of a drug discovery project allows predictions to be made regarding the feasibility of identifying a ligand that can bind with sufficient affinity to modulate the activity of the target of interest. It is important to recognize, however, that predictions made by AlphaFold will not currently account for potential conformational changes induced by PPIs, post-translational modification or small molecule binding, for example. An example of the application of AlphaFold comes from the development of a novel small molecule inhibitor of CDK20, which utilized AI technology to design small molecules based on the structures predicted by AlphaFold (126).

Amid the excitement and significant investment in AI approaches, a note of caution is that there are currently no approved drugs against targets that have been identified primarily through AI or approved drugs that have been AI designed. This is likely to change in the near future, given the recent advances in AI technology and the time taken to move through the drug discovery process. In the coming years, comparisons between costs and timelines of AI-driven approaches to conventional drug discovery will allow determination of where AI can make the biggest impact in expediting the drug discovery process.

## COMBINING DATABASE OUTPUTS FOR PRACTICAL PURPOSES

To demonstrate how outputs from a defined set of databases described within this review can be utilized in a practical setting, we use an example to illustrate how combining outputs can support evaluation of a novel target and also demonstrate how we can retrospectively analyse clinical trial failures to determine whether this could have been predicted via *in silico* analysis.

To illustrate how database outputs can support target evaluation and guide further target validation and clinical positioning efforts, we use NME6 as an example of a novel anti-cancer target. NME6 is a mitochondrial nucleoside diphosphate kinase whose primary reported function is to ensure sufficient supply of ribonucleotides to the mitochondria. A PubMed search of ‘NME6 AND Cancer’ gives only three results, none of which provide sufficient data to identify NME6 as a novel anti-cancer drug target. A recent bioRxiv preprint proposes that NME6 regulates mitochondrial gene expression and should be considered a novel target in diseases in which mitochondrial gene transcription is altered, including cancer (127).

**Figure 4.**
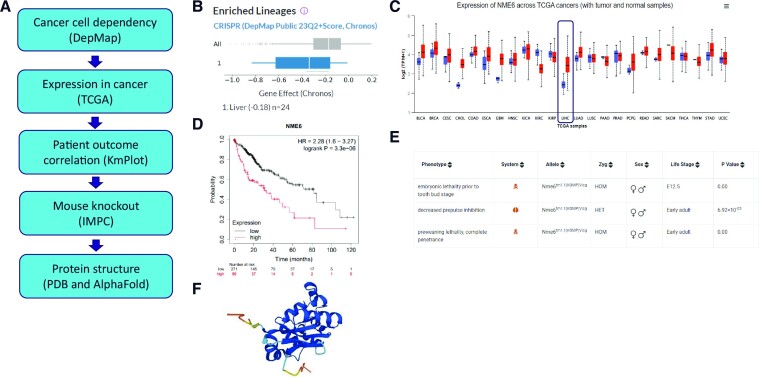
Combining databases for target evaluation. (**A**) Workflow for completing a minimal target evaluation for a novel target that has an impact on cancer cell intrinsic function. (**B**) Enriched lineage analysis from DepMap CRISPR screening data, demonstrating that liver cancer cells demonstrate a trend of increased NME6 dependence. (**C**) UALCAN portal output of patient gene expression data from TCGA comparing NME6 expression in cancer and matched normal tissue. Liver hepatocellular carcinoma is boxed for clarity. (**D**) KMplot output demonstrating patients with high NME6 have reduced overall survival compared to patients with low NME6 expression in liver hepatocellular carcinoma patients. (**E**) Summary of mouse homozygous and heterozygous NME6 knockout phenotypes from the IMPC. (**F**) Human NME6 protein structure prediction from AlphaFold (https://alphafold.ebi.ac.uk/entry/O75414).

A minimal set of databases can be used to support the unbiased evaluation of NME6 as a novel anti-cancer drug target (Figure 4A). DepMap output from CRISPR screens identified liver cancer as being the most enriched indication for cancer cell intrinsic NME6 dependency (Figure 4B). The UALCAN portal used to visualize TCGA patient gene expression data demonstrates that NME6 gene expression is increased in cancer tissue relative to adjacent normal samples in several indications, with one of the most striking upregulations being observed in liver hepatocellular carcinoma (Figure 4C; LIHC is boxed for clarity). KMplot was then used to determine whether higher NME6 expression was associated with patient outcome across 20 separate indications. There were four indications where high NME6 expression showed a significant correlation with poor outcome, with the strongest association being observed in liver hepatocellular carcinoma (Figure 4D). The IMPC mouse knockout database was used to flag adverse phenotypes associated with NME6 knockout, which may be indicative of toxicity liabilities. Homozygous NME6 knockout results in embryonic lethality, pointing to an essential role in embryonic development, while heterozygous knockout results in minimal observed phenotypes in adult mice (Figure 4E). There are no experimentally determined NME6 protein structures available within the PDB, but predicted structures are available via AlphaFold (Figure 4F). Together, this set of data provides support from cancer cell line and clinical datasets that NME6 function may be linked to liver cancer progression. Available mouse knockout data do not flag any clear toxicity liabilities associated with heterozygous knockout in the adult mouse and AlphaFold structure prediction gives a starting point for computational chemistry methods to identify putative drug-binding pockets and perform detailed ligandability assessments. Several additional databases described within this review can provide further mechanistic insight into target biology, guide the selection of clinically relevant pre-clinical model systems and guide potential NME6 targeting strategies. This minimal set of data from freely available databases addresses several of the key outputs of a target evaluation study (Figure 1), which mitigates the risk associated with committing resource to further exploration of NME6 as a novel anti-cancer drug target.

**Figure 5.**
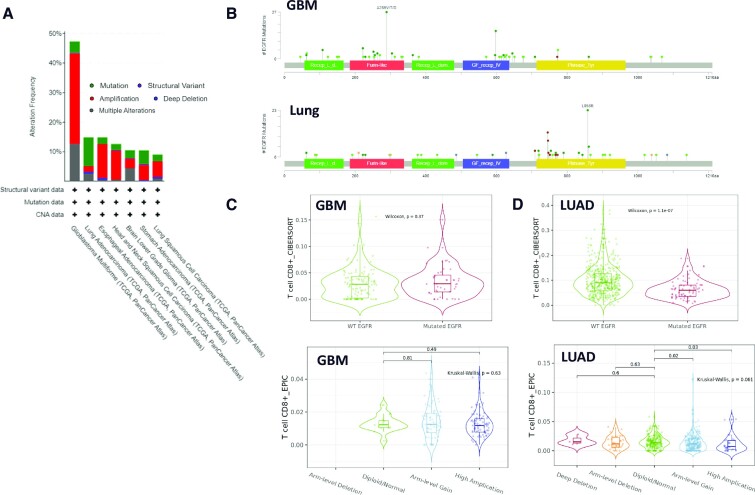
Prediction of clinical development challenges. (**A**) EGFR mutation/alteration frequency across selected cancer types from cBioPortal. (**B**) EGFR mutation landscape and properties mapped on protein domain schematic for GBM and lung adenocarcinoma (LUAD); mutations are coloured as missense (green), truncating (black), in-frame (brown), splice (orange) and fusion (purple) from cBioPortal. (**C**) Correlation between EGFR wild type and mutation (top panel) or EGFR deletion amplification versus normal (lower panel) with CD8 T-cell infiltration in GBM from TIMER2.0. (**D**) As for panel (C), with LUAD as indicated.

A key objective of the use of databases for cancer target evaluation is to reduce the high attrition rate associated with clinical development of novel compounds. As highlighted in previous sections, accurate toxicity predictions are challenging with currently available tools. However, output from the databases described within this review can inform clinical development. To illustrate how integration of database outputs could have been used to predict differing clinical outcomes between alternative indications, we evaluate epidermal growth factor receptor (EGFR) targeting in glioblastoma (GBM). cBioPortal analysis shows that EGFR gene alterations are present in approximately half of GBM patient samples, with gene amplification and mutation being the most prevalent alterations observed (Figure 5A). The high rates of EGFR alterations gave optimism that targeting EGFR could be a therapeutic vulnerability of GBM and was supported by *in vitro* data from GBM cell lines (128). However, while EGFR inhibitors have approval for treatment of EGFR mutant NSCLC, they have failed to demonstrate efficacy in GBM (129,130). Multiple reasons may account for failure in the GBM setting. The anatomical properties of the blood–brain barrier can limit drug penetrance within GBM and compensatory mutations of other receptor tyrosine kinases may drive resistance. cBioPortal analysis of mutation hotspots shows that EGFR mutations in GBM are most frequently found within the extracellular furin-like domain, while EGFR mutations in lung cancer typically occur within the intracellular kinase domain (Figure 5B). These data suggest that first- and second-generation EGFR inhibitors that target the kinase domain are not targeted towards the EGFR mutations observed in GBM and explain to some extent lack of efficacy. The efficacy of EGFR inhibition is likely to at least in part be driven by the reshaping of the tumour immune microenvironment, with higher infiltration and proliferation of anti-tumour T cells observed in mouse models following EGFR inhibition (131). GBM is well recognized as a ‘cold’ tumour type, with low infiltration of activated T cells, where the striking efficacy of immunotherapies observed in some other indications has not yet been achieved (132). Mining the TIMER database for associations between gene alteration and immune cell infiltrate illustrates that EGFR mutation or amplification in GBM patient samples does not correlate with any change in T-cell infiltration (Figure 5C), in contrast to lung adenocarcinoma, where EGFR alteration is associated with reduced T-cell infiltration (Figure 5D). Together, these database analyses predict that, despite high levels of EGFR genetic alteration observed in GBM, the lack of kinase-activating mutations and lack of impact of EGFR alteration on the tumour immune microenvironment suggest that EGFR kinase inhibitors are likely to have differing efficacy in EGFR mutant NSCLC and GBM. Such analysis may point to a need to generate compounds that target the EGFR extracellular mutation to target EGFR mutant GBM (133) or to combine with additional agents that promote increased T-cell infiltration.

## PERSPECTIVES

The breadth of free-to-use publicly available cancer databases described within this review (Table 1) allows a comprehensive evaluation of a novel target to be carried out in a short timeframe and without the need for specialist training. The key aims of target evaluation (Figure 1) are to determine whether the risks associated with a specific target programme are sufficiently mitigated by available information to support progression into the drug discovery process. Utilization of the databases described within this review can increase the information available and support assessments of existing data to inform target evaluation and consequently reduce the high attrition rate associated with novel drug development. Effective mining of databases can also be used to identify previously unknown clinical opportunities for a selected target or repurposing opportunities for an existing drug. Incorporation of patient omics data and effective utilization of clinically relevant pre-clinical models provide key data to support the development of precision medicine on a patient-specific basis.

**Table 1.** Summary table of recommended databases

Database	Content synopsis	URL
AlphaFold Protein Structure Database	AI prediction of protein structure from primary sequence with almost complete coverage of the human proteome	https://alphafold.ebi.ac.uk/
cBioPortal	Portal for exploring and analysing multi-omics characterization of patient samples	https://www.cbioportal.org/
Cancer Cell Line Encyclopedia	Genomic and metabolic characterization of cancer cell lines	https://sites.broadinstitute.org/ccle/
canSAR	Knowledgebase of multidisciplinary data that applies machine learning approaches to provide drug discovery predictions	https://cansar.ai/
Cell Model Passports	Genomic and clinical characterization of >2000 cancer cell lines	https://cellmodelpassports.sanger.ac.uk/
CellxGene	Portal to access scRNA-seq datasets	https://cellxgene.cziscience.com/
Chemical Probes Portal	Expert-reviewed online resource for identifying and using chemical probes in biomedical research and drug discovery	https://www.chemicalprobes.org
Clinical Proteomic Tumor Analysis Consortium	Houses mass spectrometry characterization of the human proteome from patient samples	https://proteomics.cancer.gov/programs/cptac
Cancer Dependency Map	Houses siRNA, CRISPR and pharmacological screening data for genomically characterized cancer cell line panels	https://depmap.org/portal/
Deeply Integrated human Single-Cell Omics data	Accesses human scRNA-seq datasets integrated into tissue-specific atlases	https://www.immunesinglecell.org/
DrugBank	Detailed information for approved drugs and investigational compounds	https://go.drugbank.com/
Genomic Data Commons data portal	Accesses TCGA multi-omics datasets from >20 000 primary cancer and matched normal samples	https://portal.gdc.cancer.gov/
Genomics of Drug Sensitivity in Cancer	Profiling of the response of >1000 cancer cell lines with over 600 approved and investigational pharmacological agents	https://www.cancerrxgene.org/
Genotype-Tissue Expression project	Gene expression data from 54 non-disease tissue types from close to 1000 individuals	https://gtexportal.org/home/
International Mouse Phenotyping Consortium	Mouse knockout phenotypic characterization from consortium aiming to knock out every protein-coding gene within mouse genome	https://www.mousephenotype.org/
Kaplan–Meier Plotter	Allows correlations between gene expression and patient outcome from manually curated datasets from several sources	https://kmplot.com/analysis/
The Human Protein Atlas	Resource that aims to map the human proteome across all major tissues and organs in normal and disease settings	https://www.proteinatlas.org/
The Mouse Models of Human Cancer Database	Knowledgebase of mouse models of human cancer with data from >46 000 models, including inbred mouse models, PDXs and GEMMs	http://tumor.informatics.jax.org
Open Targets Platform	Database for target identification and prioritization of target–disease associations	https://platform.opentargets.org/
Patient Derived Cancer Models Finder	Tool to identify suitable PDX mouse models	https://www.cancermodels.org
Probe Miner	Resource that uses fitness factors to objectively identify the best tool compounds for experimental use	https://probeminer.icr.ac.uk
PROTAC-DB	Online resource for identifying currently described PROTAC molecules	http://cadd.zju.edu.cn/protacdb/
Single Cell Expression Atlas	Portal to access scRNA-seq datasets	https://www.ebi.ac.uk/gxa/sc/home
STRING: functional protein association network	Database of known and predicted PPIs	https://string-db.org/
Structural Genomics Consortium	Portal to access information on and request chemical probes	https://www.thesgc.org/chemical-probes
TargetDB	Tool for compiling target information from public databases	https://github.com/sdecesco/targetDB
Tumor IMmune Estimation Resource	Portal to explore infiltration of immune cells in TCGA tumour samples and correlate this with gene alterations	http://timer.cistrome.org
Tumor–Immune System Interaction Database	Predicts responses to immunotherapy by integrating datasets from multiple sources, including gene expression, CRISPR/shRNA screening to determine sensitivity to T-cell-mediated killing and literature mining	http://cis.hku.hk/TISIDB/index.php
Tumor Immune Syngeneic MOuse	Syngeneic mouse model datasets including cell line genotype and cancer type, mouse genetic background and implantation site. Provides interactive visual interfaces to explore gene expression, immune cell infiltrate and response to therapy	http://tismo.cistrome.org/
University of ALabama at Birmingham CANcer data analysis portal	Houses proteomic data obtained from mass spectrometry analysis of 2002 patient samples from 17 separate studies	http://ualcan.path.uab.edu/analysis-prot.html
Worldwide Protein Data Bank	Main worldwide repository for protein structural information	http://www.wwpdb.org/

It is important, however, that database output is understood in the context of the specific limitations that we discuss throughout. For example, databases describing the impact of target depletion in cancer cell lines or in immune compromised mouse models will not be of use when predicting a target’s link to a role in the tumour immune response. Similarly, databases that house compound screening datasets will only be informative when the selectivity profile of the compounds used is known. There are also knowledge gaps that could be filled by the creation of new databases. Databases that would be of key importance may include housing datasets for cancer cell line screening under physiologically relevant cell culture conditions, a portal that allows comparison between target knockout phenotypes in cancer and non-cancer (including stromal and immune) cells, and a database that compiles *in vivo* target depletion/deletion data from tumour models.

## Data Availability

No new data were generated or analysed in support of this research. All databases used to generate data have been cited and a URL provided.
